# Heterogeneous Treatment Effects in HFpEF: Distinguishing Drug-Specific Response from Prognostic Phenotypes Across Randomized Trials

**DOI:** 10.64898/2026.07.06.26357251

**Published:** 2026-07-09

**Authors:** Clodomir Santana, Asuka Katayama, Aditya Ballal, Padmini Sirish, David A. Liem, Julie T. Bidwell, Chao-Yin Chen, Miriam Nuño, Imo Ebong, Xiao-Dong Zhang, Leighton Izu, Barry A. Borlaug, Julio A. Chirinos, Akshay S. Desai, Patrice Desvigne-Nickens, Michael M. Givertz, Sadiya S. Khan, Dalane W. Kitzman, Gregory D. Lewis, Laura J. Rasmussen-Torvik, Margaret M. Redfield, Vandana Sachdev, Svati H. Shah, Kavita Sharma, Emily Tinsley, Renee Wong, Sanjiv J. Shah, Javier E. López, Nipavan Chiamvimonvat, Martin Cadeiras

## Abstract

**Background:**

Heart failure with preserved ejection fraction (HFpEF) is a heterogeneous syndrome comprising multiple pathophysiological phenotypes. HFpEF trials have largely enrolled diverse populations and reported average treatment effects, consistently yielding neutral results that may obscure drug-specific benefits within distinct subgroups. To address this issue, we employ an interaction-based that incorporates treatment-by-variable interactions to uncover drug-specific responses.

**Methods:**

We leveraged four HFpEF clinical trials (TOPCAT, RELAX, NEAT-HFpEF, INDIE-HFpEF) and developed a framework comprising two complementary approaches. The first employed a prognostic responder model to evaluate whether conventional responder definitions reflect treatment-specific benefit or instead capture favorable clinical trajectories common to both treatment and placebo groups. The second used an interaction-based individual treatment effect (ITE) modeling to identify baseline variables that modify therapy effect, distinguishing drug-specific response from prognostic phenotypes.

**Results:**

Although the prognostic responder model demonstrated good discrimination, further analisys suggested it primarily captured a prognostic signal associated with favorable clinical trajectories common to both treatment and placebo arms. In contrast, the ITE model identified distinct, drug-specific effect modifiers across trials (cardiorenal-inflammatory for spironolactone (TOPCAT), NO-mediated anti-inflammatory for isosorbide mononitrate (NEAT-HFpEF), afterload-reducing for inorganic nitrite (INDIE-HFpEF), and anti–volume-overload for sildenafil (RELAX). Each ITE model demonstrated significance only within its own trial suggesting drug-specific signal.

**Conclusions:**

The proposed method identifies mechanism-specific effect modifiers, and uncovers clinically meaningful heterogeneity in treatment response, which is not captured by conventional MCID-based approaches. Although exploratory, these findings support phenotype-guided therapy in HFpEF and argue for phenotype-informed trial design to enhance treatment-effect detection and therapy targeting.

## Full Text Availability

The license terms selected by the author(s) for this preprint version do not permit archiving in PMC. The full text is available from the preprint server.

